# With a Little Help from My Friends: The Role of Intraoperative Fluorescent Dyes in the Surgical Management of High-Grade Gliomas

**DOI:** 10.3390/brainsci8020031

**Published:** 2018-02-07

**Authors:** Rosario Maugeri, Alessandro Villa, Mariangela Pino, Alessia Imperato, Giuseppe Roberto Giammalva, Gabriele Costantino, Francesca Graziano, Carlo Gulì, Francesco Meli, Natale Francaviglia, Domenico Gerardo Iacopino

**Affiliations:** 1 Department of Experimental Biomedicine and Clinical Neurosciences, School of Medicine, Postgraduate Residency Program in Neurological Surgery, Neurosurgical Clinic, AOUP “Paolo Giaccone”, 90100 Palermo, Italy; mariangelapino@live.it (M.P.); robertogiammalva@live.it (G.R.G.); francesca.graziano03@unipa.it (F.G.); Carlogul@yahoo.it (C.G.); gerardo.iacopino@gmail.com (D.G.I.); 2 Division of Neurosurgery, ARNAS Civico Hospital, 90100 Palermo, Italy; alessandrovilla83@gmail.com (A.V.); gabcostantino@gmail.com (G.C.); melifra75@gmail.com (F.M.); francaviglianatale@gmail.com (N.F.); 3 Division of Neurosurgery, IRCCS Neuromed, 86077 Pozzilli, Italy; alessia.imperato@gmail.com

**Keywords:** 5-aminolevulinic acid, extent of resection, fluorescein sodium, high-grade gliomas, YELLOW 560 filter, astrocytoma, glioblastoma

## Abstract

High-grade gliomas (HGGs) are the most frequent primary malignant brain tumors in adults, which lead to death within two years of diagnosis. Maximal safe resection of malignant gliomas as the first step of multimodal therapy is an accepted goal in malignant glioma surgery. Gross total resection has an important role in improving overall survival (OS) and progression-free survival (PFS), but identification of tumor borders is particularly difficult in HGGS. For this reason, imaging adjuncts, such as 5-aminolevulinic acid (5-ALA) or fluorescein sodium (FS) have been proposed as superior strategies for better defining the limits of surgical resection for HGG. 5-aminolevulinic acid (5-ALA) is implicated as precursor in the synthetic pathway of heme group. Protoporphyrin IX (PpIX) is an intermediate compound of heme metabolism, which produces fluorescence when excited by appropriate light wavelength. Malignant glioma cells have the capacity to selectively synthesize or accumulate 5-ALA-derived porphyrins after exogenous administration of 5-ALA. Fluorescein sodium (FS), on the other hand, is a fluorescent substance that is not specific to tumor cells but actually it is a marker for compromised blood-brain barrier (BBB) areas. Its effectiveness is confirmed by multicenter phase-II trial (FLUOGLIO) but lack of randomized phase III trial data. We conducted an analytic review of the literature with the objective of identifying the usefulness of 5-ALA and FS in HGG surgery in adult patients.

## 1. Introduction

Gliomas are the most common primary brain tumors in adults; they represent nearly 80% of all malignant brain tumors, with poor prognosis in their high-grade histotypes [1]. According to the current WHO grading system, high grades include grade III and IV lesions. One-year survival rate for high-grade gliomas (HGG) is 53.7%, while the two-year survival rate for these patients is only 14.6% [2]. Annually, 8000 new cases are diagnosed in the United States [3,4]. Several variables positively affect the prognosis for patients diagnosed with HGG: these include young age, tumor location, radiological features, recurrence, and the opportunity to perform an adjuvant therapy in the postoperative course [5]. Recent studies identified a strong correlation between extent of resection and overall survival with maximal survival benefit when resection volume is greater than 98% and surgery is followed by adjuvant radiotherapy and chemotherapy [6,7,8,9]. In addition to the tricky approach in head surgery [10], the similarity between tumor appearance and surrounding brain parenchyma under the operating microscope makes a complete tumor resection challenging [4]. In recent years, fluorescence-guided technology has started to emerge in glioma resection procedures to help the surgeon in differentiating intraoperatively neoplastic tissue from normal brain in order to maximize the extent of glioma resection. Several fluorescent biomarkers have been investigated to improve intraoperative identification of residual tumor: 5-aminolevulinic acid (5-ALA) and fluorescein sodium (FS) are the most promising in glioma surgery [11,12].

## 2. Materials and Methods

### Search Strategy

Our research of the PubMed database was initiated on 1 June 2017 with the aim of identifying all studies related to the use of 5-ALA and FS in HGG surgery. We performed the literature review on the PubMed database using the following combinations of terms: “aminolevulinic acid” AND “brain neoplasms”, “aminolevulinic acid” AND “brain tumor”, “aminolevulinic acid” AND “glioma”, “5-ALA” AND “brain neoplasm”, “5-ALA” AND “brain tumor”, “5-ALA” AND “glioma”, “fluorescein sodium” AND “brain neoplasms”, “fluorescein sodium” AND “brain tumor”, “fluorescein sodium” AND “glioma”. We selected only articles written in English language. Retrospective, prospective studies, and clinical trials were included, while editorials, case reports, and commentaries were excluded. 

The results of the literature review were categorized according to the main topic they dealt with to distinguish between two categories: (1) articles dealing with the analysis of the accuracy 5-ALA of and FS in high-grade gliomas, and (2) articles concerning the clinical efficacy and safety of the 5-ALA and FS in HGG surgery.

## 3. Results

### 3.1. 5-ALA

5-Aminolevulinic acid (5-ALA) represents the precursor of heme synthesis pathway, which leads to the production of protoporphyrin IX (PpIX). This is a molecule able to emit fluorescence when excited by appropriately filtered light [13]. Under blue-violet light conditions, the PpIX emits light in the red region of the visible spectrum, enabling identification of tumor tissue that would otherwise be difficult to distinguish from normal brain [14].

Malignant glioma cells have the capacity to selectively synthesize or accumulate 5-ALA-derived porphyrins after exogenous administration of 5-ALA. To use fluorescence-guided surgery, the surgical microscope is provided by a xenon light source; this permit to switch between white bright and the violet-blue light (370–440 nm) needed to excite PpIX [15]. In order to visualize the tumor as a red fluorescent mass (emission peaks between 635 and 704 nm), a proper filter is added to the microscope lens [13,16].

It has been demonstrated that 5-ALA is safe and well-tolerated; the main side effect is sensitization of the skin that lasts for 24 h. Direct sunlight or strong room light exposure need to be avoided: therefore, during the surgical procedure patients should be shielded from overhead lights by drapes [17].

Since the introduction of 5-ALA, many studies have reported the effective role of this dye in HGG surgery.

First of all, in 1998, a direct correlation between the intraoperative macroscopic detection of fluorescence given by porphyrin and the malignant glioma tissue was demonstrated by Stummer in a series of patients given 10 mg 5-ALA/kg body weight after the operating microscope had been modified. Thanks to the guidance of fluorescence within the tumor cavity, the resection of residual tumor was possible in seven of nine patients. In these cases, after the resection, no residual fluorescence was intraoperatively observed and post-operative magnetic resonance imaging (MRI) scans did not show any residual tumor [18]. The accumulation of 5-ALA and PpIX in malignant cells is explained by many theories. Increased permeability of blood brain barrier, increased uptake of PpIX by adenosine triphosphate (ATP)-binding cassette B6, reduction of ferrochelatase levels, increased metabolism of malignant tumor cells, elevated cellular density, neoangiogenesis in areas of malignancy have all been implicated [17].

The incidence of erroneous results in fluorescence tissue with 5-ALA is low and still under study.

False-positivity is debated, Masubuchi et al. suggested that 5-ALA may be converted in PpIX by the tumor cells and then secreted in the extracellular space [19]. Another theory involves 5-ALA leakage from the tumor into surrounding tissue because of disruption in the blood brain barrier (BBB) where it is converted into PpIX [13]. Reactive astrocytosis areas, fibrotic, high vascularized tissues or inflamed tissues with macrophages infiltration may emit fluorescence even if in absence of malignant cells. Diffuse and moderate fluorescence may be emitted also by specific brain structures, such as choroid plexus, ependyma and pia mater [20].

False negative can be determined by specific conditions: first of all, cancer cells may be found even far from the contrast-enhanced tumor mass because of infiltrative growth of malignant gliomas, therefore reducing local fluorescence. Secondly, lack of fluorescence can be due to structural obstacles limiting the visualization of fluorescence, such as blood or overhanging healthy brain tissue obstructing the view of the resection cavity or inadequate illumination by the blue light from the microscope. Third, an inadequate administration timing of 5-ALA may also cause the lack of tumor fluorescence in limited cases. This can happen if the patient undergoes too early to surgery (less than 2 h) or too late after oral administration of 5-ALA [16].

### 3.2. 5-ALA and Intensity of Fluorescence

Díez Valle et al. [21] analyzed the efficacy of 5-ALA fluorescence in a series of 36 consecutive patients with glioblastoma multiforme (GBM). In those cases, the dissection was planned by the use of the fluorescent light in order to point out the right dissection plane. In each case, histological investigations by the use of hematoxylin-eosin coloration and immunostanding were performed on several samples with different fluorescent quality, collected from the center of the tumor, from the edges and from the surrounding tissue. Solid tumor was definitively identified by solid fluorescence whit 100% positive predictive value (PPV). Vague fluorescence identified the invaded tissue beyond the tumor mass with 97% positive predictive value and 66% negative predictive value (NPV) [15].

Stummer et al. in a clinical series of 52 patients affected by glioblastoma multiforme (GBM), distinguished two distinct qualities of fluorescence: solid as red and vague as pink. Red fluorescence was observed in highly perfused tissue with neoangiogenesis found on histological examination, while pink fluorescence was observed in the peritumoral tissue (infiltrating tumor cells or edematous brain tissue). Necrotic degenerated tissue showed no fluorescence. In both fluorescence types, PpIX was found in the cytoplasm of tumor cells [13,22].

Surgeon’s subjective perception of the fluorescent appearance of the lesion under white light was assessed by Nabavi and colleagues. In a multicenter prospective, single-arm, uncontrolled phase II study, 36 patients with WHO grade II/IV recurrent glioma were given 5-ALA before surgical procedure. After microsurgical resection, biopsies from pathological and non-pathological areas (as identified under conventional white light) were obtained to determine the positive predictive value (PPV) of 5-ALA—induced tissue fluorescence in detecting tumors. PPV was defined as the percentage of patient showing positive tumor cell identification in all biopsies. As showed by collected data, patient PPV was 97.2% for pathological areas and 79.4% in non-pathological areas. In this way fluorescence showed a high PPV for tumor tissue, even if it looks like normal tissue under white light, thus being effective for the detection of early recurrences [23].

### 3.3. 5-ALA and Extent of Resection

5-ALA influenced most of all dyes the resection of primary malignant brain tumors in the last few years. Its use determined a higher resection rate, which led to a longer progression free survival and a longer overall survival.

In a recent meta-analysis by Eljamel, a gross total resection rate (removal of 98% or more of the contrast-enhancing tumor) of 75.4% (418/565 patients) was achieved in GBM patients after 5-ALA glioma surgery [24].

In the prospective study of Stummer et al. [22] it has been possible to achieve complete resection with the complete removal of fluorescent tissue in 17 of 52 patients with GBM. Gross resection with some left fluorescent tissue was achieved in the other 35 patients. Despite the surgical detection, on postoperative magnetic resonance (MR) images complete resection of contrast-enhancing tumor was observed in 33 (63%) of 52 patients. Among the reasons that might have influenced the sub-total instead of total resection, tumor closeness to eloquent areas was the only factor that independently influenced the resection rate. This was assessed by the analysis of residual contrast-enhancing volume of neoplastic tissues on post-operative MRI [22].

Similarly, in 2006 a randomized controlled trial by Stummer et al. [25] evaluated the extent of resection using 5-ALA compared to standard white light in 322 patients with suspected GBM. A total of 139 patients received fluorescence-guided resection, while 131 had standard microsurgical resection. In the fluorescent-guided surgical group, gross total resection was achieved in 90/139 (65%) of the patients, while a complete resection rate of 36% (47/131) was achieved in the with light resection group. These results demonstrated that, 5-ALA can enhance total resection rate for high-grade gliomas compared to white light [25].

Other studies validated these results; Díez et al. achieved complete resection of contrast-enhancing volume in 83.3% of 36 patients affected by GBM [15,25]. Similarly, in the retrospective studies of Schucht et al. and Della Puppa et al. always 5-ALA allowed with high gross total resection (GTR) rates [26,27,28].

### 3.4. 5-ALA and Intraoperative Tools

It has been shown by several studies that 5-ALA with other intraoperative equipment such as neuronavigation, monitoring, mapping or intraoperative MRI, may enhance the rate of complete resections in high-grade gliomas.

The combination of 5-ALA and neuronavigation was studied by Panciani et al. in a prospective study of 18 patients with presumed GBM. Fluorescence-guided resection showed a sensitivity of 91.4% and a specificity of 89.2%. The use of neuronavigation improves the sensitivity up to 97.2% while reduces specificity to 45.9% because of by peritumoral inflammation that increased mitotic activity. According to the authors, the combination of fluorescence and neuronavigation may improve the detection of pathological tissue not visible with conventional surgical strategy [29].

To the best of our knowledge very few studies investigated the role of HGG surgery in eloquent areas combining 5-ALA and intraoperative monitoring. The achievement of gross total resection was reported by Feigl et al. in 64% of 18 patients treated with a combined strategy. The resection was stopped in 24% of cases, because of the identification of functional area or a cortical tract. The reported morbidity was 12% [30].

In a series of 53 HGG patient eligible for gross total resection (GTR) or complete resection of enancing tumor (CRET), Schucht and colleagues reported a complete resection of enhancing tumor in 89% (47 of 53 patients), 97% (33 of 34) of those cases preoperatively presumed to be in non-eloquent location, and 74% (14 of 19) of those presumed to be in eloquent location. These data showed that 5-ALA guided surgery together with intraoperative mapping improves rate of CRET and GTR [28].

The use of 5-ALA—guided surgery combined with updated intraoperative imaging information, such as iMRI, has been investigated in several studies. In a prospective study by Coburger et al. 45 patients with contrast-enhancing lesions underwent surgery finalized to GRT. In all procedure, surgery was guided by iMRI and 5-ALA for all patients according to a specific protocol. First, a standard tumor removal was performed under white light. Then, residual tumor location was marked by fluorescence and iMRI. Navigated biopsy samples were also taken from all marked areas. 34 patients with HGGs were enrolled. Data showed that in HGG, 5-ALA showed a higher sensitivity for tumor detection (0.85) than iMRI (0.41). Specificity was significantly lower in 5-ALA (0.43) than in iMRI (0.70). For detection of pathological tissue, 5-ALA significantly exceeded iMRI in specificity (0.80 vs. 0.60) and sensitivity (0.91 vs. 0.66) (*p* < 0.001). As regard the appearance in HGG surgery, 5-ALA and iMRI showed different sensitivity at the border zone; 5-ALA has a higher sensitivity and a lower specificity for tumor detection than gadolinium-diethylenetriamine pentaacetic acid (Gd-DTPA)-enhanced iMRI. For detection of infiltrating tumor at the border of the resection cavity, 5-ALA is superior to Gd-DTPA-enhanced iMRI concerning both sensitivity and specificity [31].

The combination of 5-ALA, iMRI and neuronavigation was investigated by Yamada et al. in a series of 98 patients with HGGs [20]. According to contrast-enhanced iMRI, several specimen of tumor tissue were taken from the bulk of the tumor. Those specimens showed strong (91 cases), weak (6 cases), or absent (1 case) fluorescence. Therefore, in such cases tissue sampling based on the anatomical information alone had 100% of Positive Predictive Value (PPV) for glioma presence. All tissue specimens (*n* = 188) were obtained from the “peritumoral brain” according to contrast-enhanced iMRI. In those specimena histopathological investigation revealed neoplastic elements in 143 cases (76%). According to these data, removing the bulk of the tumor may be effectively performed by neuroimaging alone, since it provided 100% PPV for tumor presence in the resected pathological material. In the same time, the use of the 5-ALA allows more radical tumor resection since its fluorescence extends beyond radiological margins of the neoplasm [20].

Nevertheless Roder et al. contradict these results since no advantage by the fluorescence was found in HGG surgery in comparison with iMRI-guided resection of HGG. Moreover, their results showed that iMRI is superior to 5-ALA regarding to residual tumor and total resection rate, particularly in those lesions involving eloquent brain areas [32].

On the other hand, several other studies confirmed that the combination of intraoperative MRI and 5-ALA has a synergistic effect in glioma surgery [33,34] also with functional neuroimaging.

In a series of 36 HGG patients undergoing surgical resection aided by 5-ALA and functional neuroimaging (functional MRI (fMRI) and diffusion tensor imaging (DTI)), Eyupoglu et al. performed more radical resection within functional limits thanks to the revelation of residual enhancement on iMRI. Combining both functional imaging and 5-ALA fluorescence was especially effective in tumors involving brain areas and increased the extent of resection in this subgroup from 71.7 to 100% [35].

### 3.5. 5-ALA and Outcome

In the previous mentioned randomized controlled trial involving 322 HGG patients, Stummer et al. analyzed the role of 5-ALA on extent of resection, progression-free survival (PFS), OS, and morbidity, comparing 5-ALA-guided to conventional white-light resection. Better results were achieved by the use of 5-ALA guided resection, with the performing of CRET in 90 of 139 (65%) patients in the 5-ALA group compared with 36% (47 of 131) in the control group. The median survival of CRET patients was 17.9 months compared to 12.9 months of the control group. Moreover, a 6-months PFS was more widely obtained in 5-ALA group (41.0% in the 5-ALA group vs. 21.1% in white-light group). The median PFS was 5.1 months in the 5-ALA group vs. 3.6 months in the control group. 

In a further study by the same group it has been demonstrated, in a series of 52 GBM patients undergone surgical resection with 5-ALA, that survival was related to the residual fluorescence at the end of surgery (no residual fluorescence: mean survival 101 weeks; residual vague fluorescence: 79 weeks; residual solid fluorescence: 51 weeks). At the same way, survival was also related to residual contrast enhancement on postoperative MRI (no residual contrast enhancement: 103 weeks; residual contrast enhancement: 54 weeks) [17,22,25].

These data have been validated by several studies in which it has been confirmed that 5-ALA decreases residual tumor volume and can improve GRT rate in HGGs, with a significant improvement of the overall survival [36,37]. 

Literature clinical series have been summarized in Table 1.

Fluorescein sodium (FS) is a fluorescent substance that can be used for immediate improved visualization of brain tumor tissue, with no specificity for tumor cells [52]. This dye, if excited by a light whose wavelength is in the range of 460–500 nm, emits fluorescent radiation with wavelength range of 540–690 nm. Since fluorescein sodium is not taken by astrocytoma cells, but it can be accumulated in extracellular tumor areas, it has been suggested that it can mark for compromised blood-brain barrier areas, as in high-grade astrocytomas. Widely used in ophthalmic surgery, fluorescein is injected intravenously just before glioma resection, it is virtually free of side effects and has low costs, approximately 5 euros each vial (1 g of substance) [53,54]. It is usually visible to the naked eye at high dosage (20 mg/kg body weight) and at lower doses, it is observable under the YELLOW 560 nm surgical microscope filter, allowing better tissue discrimination with natural colors [12,55,56].

Fluorescein sodium is a fluorophore that has been used in medical applications for more than six decades [57]. This dye penetrates in those areas of the brain where the blood-brain-barrier (BBB) is damaged, allowing real time enhancement of the areas contrasted by gadolinium in MRI. In experimental studies, rodent BBB disruption with intra-arterial mannitol administration has been demonstrated to enhance the fluorescein signal in the brain [58]. The fluorescence of FS can be grossly perceived to the naked eye, when this agent is injected at high doses (20 mg/kg). However, when using a recent developed microscope integrated YELLOW 560 module (Carl Zeiss Meditec, Oberkochen, Germany), we can employ a low dose of FS (5 mg/kg) to detect an optimal fluorescence within the tumoral tissue [59].

Although widely accepted in several fields of medicine as a very safe molecule, side effects of fluorescein administration, as skin reactions, syncope, respiratory or cardiac adverse effects, and seizures have been reported [60].

From the analysis of the literature it emerges that, besides its wide and safe use in ophthalmology [4], fluorescein injection seems to be a good method to obtain a high rate of gross total resection during malignant brain tumors surgery [61]. The percentage of resection in the series that we have analyzed varied from 75 to 100% [62].

In 2003 Shinoda et al. reported a series of 32 patients surgically treated for glioblastoma multiforme. In 84.4% of these patients, gross total removal was obtained with a high dose of fluorescein sodium (20 mg/kg bodyweight). Only 30.1% obtained GTR with conventional white light imaging; no difference in the overall prognosis was observed in this series [63].

In 2008 Koc et al. reported a prospective non-randomized study to evaluate the influence of fluorescein sodium-guided glioma resection on gross total tumor removal (GTR), overall prognosis, and side effects. A high intravenous dose (20 mg/kg body weight) was administered to 47 out of the 80 enrolled patients after craniotomy. A standard operating room microscope without a filter or special camera was used. A second group of 33 patients underwent ordinary resections. A significant increase in the GTR rate (83% vs. 55%) was achieved whit the administration of fluorescein. No statistically significant difference in overall survival between the two groups (44 vs. 42 weeks) was observed [4,64].

In 2012 Chen, in a cohort of 22 patients with HGGs, observed a significant improvement in progression-free survival, together with the GTR rate, in patients treated with the aid of intraoperative intravenous injection of fluorescein sodium (15–20 mg/kg bodyweight) compared to the control group’s progression-free survival [2].

In the same year, Okuda reported the safety and the efficacy of a new technique of fluorescence-guided surgery for GBM surgery based upon high dose fluorescein sodium with excitation and barrier filters on the operating microscope (OME-9000 Olympus, Olympus Medical Systems Corp., Tokyo, Japan). This new technique was employed in a series of 10 patients, enabling a detailed tumor assessment and allowing the identification of tumor vessels and surrounding normal vessels. This dye allowed performing the surgical removal with both fluorescence and under normal white xenon-light illumination [52].

After the study of Kuroiwa, who was the first to describe a novel technique of integration of the fluorescence filter in the microscope (Zeiss) [60], Schebesch in 2013 published data about a series of 35 patients with malignant brain tumor (whose 22 WHO HGGs including 5 recurrent glioblastomas) surgically treated with the aid of a reduced dose (3–4 mg/Kg) of FS and with an intraoperative PENTERO 900 microscope equipped with the 560-nm wavelength fluorescence light filter. In all cases, the tissue fluorescence was brightly visible 30 min after administration of FS and it lasted for the entire duration of the procedure, representing a significant and “helpful” mean for the surgeon in 28 out of 35 cases [65].

Finally, Acerbi et al. was the first group to initiate a prospective Phase II trial (FLUOGLIO) in 20 consecutive patients with HGGs. In these patients, fluorescein sodium was administered intravenously at the induction of anesthesia. In this case, the fluorescence visualization during the surgical procedure was obtained with BLUE 400 or YELLOW 560 filters on a PENTERO 900 microscope at a very low dose (10 mg/kg with the BLUE 400 filter and at 5 mg/kg with the YELLOW 560 filter). Data revealed a complete resection of the tumor in 80% of patients, a 6-months progression-free survival rate for 71.4%; moreover, a median survival of 11 months and a median duration of follow up of 10 months were shown [53,62,66,67].

In February 2016, Hamamcıoğlu et al. presented their series of 23 high-grade tumors and 7 metastatic tumors treated with the intraoperative aid of 200 mg (2–4 mg/kg) of fluorescein sodium. This dye was found “helpful” for tumor demarcation in 29 out of 30 operations (97%). In 23 of these 29 operations (79%), a total resection (radiologically demonstrated) was achieved regardless of the histopathology, whereas a near-total resection was achieved in 4 patients (14%). A subtotal resection was achieved in the remaining two patients (7%) [68].

In 2017 Francaviglia et al. reported a gross total resection in 53.2% (*n* = 25) of patients with high-grade gliomas. A subtotal resection (>95%) was achieved in 29.8% (*n* = 14) of them, while a partial resection (<95%) was obtained in 17% (*n* = 8) of patients. Globally, a resection >95% was achieved in 83% (*n* = 39) of patients who underwent fluorescence-guided surgery. The median postoperative KPS score was 83.4 (range: 50–100). Comparing pre- and post-operative scores, the latter was found higher in 15 patients, lower in 11 and stable in 21 patients [69].

On the other hand, Schwake et al. conducted a small pilot study using two timing regimes in four patients pre-treated with 5-ALA (20 mg/kg, Gliolan; Medac, Wedel, Germany), comparing early (35 min prior to durotomy, as previously described [65]) with acute injections of fluorescein (4 mg/kg, Fluorescein Alcon 10%; Alcon, Freiburg, Germany). They observed diffuse fluorescence of fluorescein along resection margins spreading through the arachnoid space away from the lesion. Overall, they observed no clear value of fluorescein in their small study, which they closed prematurely [12].

Literature clinical series have been summarized in Table 2.

It is clear that the use of intravenous fluorescein during the surgical removal of high-grade gliomas with a specific yellow filter, may be a very effective, safe, and inexpensive way to achieve a gross total removal of the tumor. Nevertheless, it should be emphasized that fluorescence technology shows blood brain barrier breakdown, corresponding to the areas marked by the contrast agent in MRI. Consequently, a resection based on FS fluorescence, allows the removal of those neural tissue portions in which the BBB is interrupted. It does not necessarily allow resection of the full extent of infiltrating tumor cells, thus potentially reducing the accuracy of tumor identification. Moreover, the necrotic portion of the tumor does not stain with fluorescein, due to its lack of cerebral vasculature [55,67].

## 4. Conclusions and Future Perspectives

Several fluorescent agents have been investigated for use in glioma surgery to improve intraoperative identification of tumors. 5-aminolevulinic acid (5-ALA) and fluorescein sodium (FS) are the most promising and the object of our study but also indocyanine green (ICG), hypericin, 5-aminofluorescein labeled to human serum albumin (AFL-HSA), and endogenous spectroscopy have been tested clinically in the literature [11,71].

The ideal fluorescent agent in fluorescence-guided surgery should be safe to use, easy to apply, tumor-specific, with a strong and easy signal to detect. Currently, 5-ALA is the only agent that has been tested in a multi-center randomized controlled trial and has been approved for clinical use in certain countries of the world. 

The randomized controlled trial we have analyzed demonstrated that the use of 5-ALA-based fluorescence-guided surgery (FGS) results in improved extent of resection in FGS for glioma [25]. Observational cohort studies suggest that also fluorescein sodium increases the rate of GTR [2,63,64,69], and it has a positive influence on PFS [2].

Although specificity and sensitivity for tumoral tissues of 5-ALA are 100% and a 85% respectively [52], various elements have limited its use in fluorescence-guided surgery: first of all its elevated cost when used from the EMEA approved manufacturer—Gliolan (almost 1000 euros for each vial)—but also the oral route of administration some hours before the induction of anesthesia with the need to wait for the peak concentration in the blood in order to visualize fluorescent tissue; furthermore it requires a dark surgical field not always comfortable for the operator; finally with 5-ALA usage there is a high risk of skin sensitization within 24 h after the operation (the patient should not be exposed to sunlight or artificial light) [53].

On the contrary, fluorescein sodium is a substance that can be used for immediate improved visualization of tumor, despite its non-specificity for tumor cells, for compromised blood-brain barrier (BBB) areas [52]. FS is injected intravenously just after the induction of general anesthesia, it is virtually free of side effects and has low costs (approximately 5 euros each vial: 1 g of substance) [53,54]. At high dosage (20 mg/kg body weight) FS is visible to the naked eye while at lower doses, it is observable under the YELLOW 560 nm surgical microscope filter. The tumor is visible as bright yellow tissue different from the surrounding brain; the tissue discrimination is possible with more natural colors than with 5-ALA [12,55].

To date, the evidence for effectiveness of fluorescein sodium has been based only on observational cohort studies and case series. Currently, a direct comparison between 5-ALA and fluorescein sodium is therefore not possible and would require specifically designed studies.

Well-designed randomized controlled trials to evaluate fluorescein sodium safety and effectiveness are essential. 

## Figures and Tables

**Table 1:** Clinical series of patients with gliomas treated with a 5-amino-levulinic acid aided surgery. Literature review.

Author and Year	Study Design	N° Pts	Tumor Type (*n*)	GTR Rate (%)	Sensitivity %	Specificity %	Primary Endpoint
Stummer et al., 1998 [18]	CT	10	Malignant gliomas:	7/10 (70%)			To evaluate the use of 5-ALA in patients with malignant gliomas
			Grade III (2)		87 (Grade IV)	100 (Grade IV)	
			Grade IV (8)		71 (Grade III)	100 (Grade III)	
Stummer et al., 2000 [22]	Pros	52	GBMs	33/52 (63%)	/	/	To evaluate the impact of 5-ALA guided surgery on GTR, postoperative MRI findings and survival
Stummer et al., 2006 [25]	CT	139	Malignant gliomas:	90/139 (65%)	/	/	To assess GTR rate, survival (OS and PFS), adverse events using 5-ALA guided resection vs. controls
			Grade III (4)				
			Grade IV (135)				
Nabavi et al., 2009 [23]	Pros	36	Recurrent malignant gliomas:	7/36 (19.4%)	82	97	To assess the applicability of 5-ALA—resection for recurrent malignant gliomas
			Grade III (13)				
			Grade IV (21)				
			Secondary GBMs (2)				
Widhalm et al., 2010 [38]	Pros	17	Diffusely infiltrating gliomas:	14/17 (82%)		/	Clarify whether 5-ALA may visualize anaplastic foci in diffusely infiltrating gliomas with non-significant contrast enhancement.
			Grade II (8)		0 (Grade II)		
			Grade III (9)		89 (Grade III)		
Díez Valle et al., 2011 [21]	Pros	36	Malignant Gliomas:	30/36 (83.3%)	47	100	To evaluate the diagnostic accuracy, GTR rate, and safety, of 5-ALA guided surgery
			Newly diagnosed (28)				
			Recurrent GBMs (8)				
Feigl et al., 2010 [30]	Pros	18	Malignant Gliomas:	16/25 (64%)	/	/	To evaluate the utility and safety of combining 5-ALA—guided resection of malignant gliomas in eloquent areas and intraoperative neurophysiological monitoring.
			Grade III (3)				
			Grade IV (15)				
Roberts et al., 2011 [39]	Pros	11	Newly diagnosed GBMs	/	75	71	To investigate the relationships between intraoperative fluorescence, features on MR imaging, and neuropathological parameters.
Takahashi et al., 2011 [40]	Pros	19	Malignant brain tumors:	/		/	Evaluate the molecular mechanisms underlying PpIX accumulation in clinical malignant brain tumors following administration of 5-ALA.
			GBMs (9)		78 (GBMs)		
			Metastases (10)		30 (Metastases)		
Tsugu et al., 2011 [34]	Retro	33	Gliomas:	6/11 (54.5%) only 5-ALA; 4/10 (40%) with iMRI		/	Evaluated intra-operative MRI—guided resection combined with resection guided by 5-ALA—fluorescence.
			Grade II (6)		0 (grade II)		
			Grade III (7)		57 (grade III)		
			Grade IV (20)		85 (grade IV)		
Valdes et al., 2011 [41]	CT	23	Gliomas:	/		/	To evaluate whether quantitative ex vivo tissue measurements of 5-aminolevulinic acid induced PpIX identify regions of increasing malignancy in low- and high-grade gliomas.
			Grade I (4)		0 (grade I)		
			Grade II (2)		50 (grade II)		
			Grade III (3)		100 (grade III)		
			Grade IV (12)		100 (grade IV)		
Schucht et al., 2012 [28]	Retro	53	Newly diagnosed and recurrent GBMs	51/53 (96% of GTR-eligible patients)	100	/	To evaluate the efficacy and safety of the association of 5-ALA and functional mapping for surgery of GBMs.
Tykocki et al., 2012 [42]	Small Case Series	5	Malignant Gliomas:	4/5 (80%)	80	/	To evaluate the efficacy and safety of 5-ALA—guided resection of malignant gliomas.
			Newly diagnosed (2)				
			Recurrent GBMs (3)				
Della Puppa et al., 2013 [26]	Pros	31	Malignant Gliomas:	23/31 (74%)		/	To evaluate the efficacy and safety of the association of 5-ALA and functional mapping for surgery of malignant gliomas HHG in eloquent areas.
			Grade IV (25)		100 (grade IV)		
			Grade III (6)		100 (grade III)		
Widhalm et al., 2013 [43]	Pros	59	Gliomas with no significant contrast-enhancement:	38/59 (64%)	89 (to detect anaplastic histology)	88 (to detect anaplastic histology)	To evaluate whether 5-ALA might serve as marker for visualization of anaplastic foci in diffusely infiltrating gliomas with non-significant contrast enhancement.
			Grade II (33)				
			Grade III (26)				
Coburger et al., 2014 [31]	Pros	42	High grade gliomas and metastasis:	/			To evaluate whether 5-ALA fluorescence provides an additional benefit in detection of invasive tumor compared with intraoperative MRI (iMRI).
			Grade III-IV (34) Metastases (8)		Solid tumors: 85 (grade III-IV) 75 (metastases) Infiltrating tissue: 91 (grade III-IV) 88 (metastases)	Solid Tumors: 43 (grade III-IV) 80 (metastases) Infiltrating tissue: 80 (grade III-IV) 75 (metastases)	
Marbacher et al., 2014 [33]	Retro	458	Intracranial tumors:	/		/	To evaluate the safety and clinical utility of 5-ALA in resection of brain tumors other than glioblastomas.
			Grade I/II (20)		40 (Grade I-II)		
			Grade III/IV (138)		88 (Grade III-IV)		
			Meningiomas (110)		85 (meningiomas)		
			Metastases (75)		52 (metastases)		
Stummer et al., 2014 [44]	Pros	33	Malignant Gliomas:	/	/	/	To determine the value of visible fluorescence qualities “strong” and “weak” for predicting tissue morphology.
			Grade III (4)				
			Grade IV (29)				
Yamada et al., 2015 [20]	Pros	99	Malignant Gliomas:	51/99 (52%)	95%	53%	To evaluate the role of 5-ALA during intraoperative MRI guided resection.
			Grade III (32)				
			Grade IV (67)				
Jaber et al., 2016 [45]	Pros	166	Gliomas:	/	/	/	To identify preoperative factors for predicting fluorescence in gliomas without typical GBM imaging features.
			Grade II (82)				
			Grade III (76)				
			Grade IV (8)				
Haj-Hosseini et al., 2015 [46]	Pros	30	Malignant Gliomas:	/	/	/	To investigate the use of lower 5-ALA dose (5 mg/kg) compared with higher dose (20 mg/kg).
			Mixed grade III-IV (1)				
			Grade III (2)				
			Grade IV (27)				
Teixidor et al., 2016 [47]	Pros	77	Malignant Gliomas:	41/77 (53.9%)	/	/	To evaluate the effectiveness and safety of 5-ALA.
			Grade III (11)				
			Grade IV (66)				
Szmuda et al., 2015 [48]	Pros	21	Malignant Gliomas:	/	/	/	To reveal the shortcomings of 5-ALA fluorescence perception by surgeon’s eye in order to direct further improvements in image filtering and digital processing.
			Grade III (2)				
			Grade IV (19)				
Chan et al., 2017 [49]	Retro	16	Suspected Malignant Gliomas:	9/16 (56.2%)	/	/	To evaluate the percentage of patients who had brain tumors totally excised under the guidance of 5-ALA.
			Grade I-II (3)				
			Grade III (2)				
			Grade IV (10)				
			Others (1)				
Cozzens et al., 2017 [50]	CT	19	Metastatic lung adenocarcinoma (1); Grade III (2) and Grade IV (16) gliomas	11/19 (57.9%)	/	/	To identify the appropriate dose and toxicity of 5-ALA used for enhanced intraoperative visualization of malignant brain tumors.
Saito et al., 2017 [51]	Retro	60	Gliomas:	/	/	/	To analyze factors (bimolecular, imaging and histological findings) influencing the intraoperative visualization of gliomas by their 5-ALA-induced fluorescence.
			Grade II (8)				
			Grade III (17)				
			Grade IV (35)				

Abbreviations: 5-ALA = 5-amino-levulinic acid, / = not reported, CPOX = Coproporfirinogen Oxidase, CT = controlled trial, GBM = glioblastoma multiforme, GTR = gross total resection, MRI = magnetic resonance imaging, *n* = number, NPV = negative predictive value, OS = overall survival, PET = positron emission tomography, PFS = progression-free survival, PpIX = protoporphyrin IX, PPV = positive predictive value, Pros = prospective, Pts = patients, Retro = retrospective fluorescein sodium.

**Table 2:** Clinical series of patients with gliomas treated with a fluorescein sodium aided surgery. Literature review.

Author and Year	Study Design	N° Pts	Tumor Type (*n*)	GTR Rate (%)	Sensitivity %	Specificity %	Primary Endpoint
Kuroiwa et al., 1998 [60]	Retro	10	Malignant Gliomas: Grade III (5) Grade IV (5)	8/10 (80%)	/	/	To evaluate the efficacy of FS in malignant gliomas.
Shinoda et al., 2003 [63]	Retro	32	GBMs	27/32 (84.4%)	/	/	To evaluate the efficacy of FS in GBMs without any special surgical microscopes.
Koc et al., 2008 [64]	Pros	47	GBMs	39/47 (83%)	/	/	To evaluate the influence of FS-guided resection on GTR and overall survival in a series of patients with GBM.
Chen et al., 2012 [2]	CT	10	Gliomas: Grade II (4) Grade III (3) Grade IV (3)	8/10 (80%)	/	/	To reevaluate the utility and clinical limitations of using fluorescein sodium for the treatment and resection of glioma brain tumors.
Schebesch et al., 2013 [65]	Retro	35	Brain tumors: Grade I (1) Grade II (3) Grade III (5) Grade IV (17) Metastases (5) non-malignant astrogliosis (2) post-radiation necrosis (2)	28/35 (80%)	/	/	To assess the feasibility and efficacy of FS under YELLOW 560 nm.
Acerbi et al., 2014 [67]	Pros	20	Malignant Gliomas: Grade III (1) Grade IV (19)	16/20 (80%)	94%	89.5%	To evaluate the safety of fluorescein-guided surgery for HGGs and obtaining preliminary evidence regarding its efficacy for this purpose.
Diaz et al., 2015 [55]	Pros	12	Malignant Gliomas: Newly diagnosed GBMs (9) Recurrent GBMs (3)	12/12 (100%)	82.2%	90.9%	To assess the intraoperative application of this technology.
Hamamcıoğlu et al., 2016 [68]	Retro	29	Malignant brain tumors: Grade III (6) Grade IV (15) Metastases (6) CNS lymphomas (2)	23/29 (79%)	/	/	To confirm that FS guidance with the use of YELLOW 560 nm filter is safe and effective in high-grade glioma and metastatic tumor surgery.
Catapano et al., 2017 [70]	Retro	23	Malignant Gliomas: Grade III (1) Grade IV (22)	19/23 (82.6%)	84.6%	95%	To contribute to the investigation according to which FS-guided surgery for HGG is related to better rates of GTR.
Francaviglia et al., 2017 [69]	Retro	47	Malignant Gliomas: Grade III (14) Grade IV (33)	39/47 (83%)	/	/	To assess the role of FS in achieving GTR and in distinguishing tumoral by normal brain tissue.

/ = not reported, CT = controlled trial, GBM = glioblastoma multiforme, GTR = gross total resection, Pros = prospective, Pts = patients, Retro = retrospective.

## Abbreviations

HGG	high-grade gliomas
PFS	progression-free survival
OS	overall survival
5-ALA	5-aminolevulinic acid
FS	fluorescein sodium
PpIX	protoporphyrin IX
EOR	extent of resection
GTR	gross total resection
PPV	positive predictive value
NPV	negative predictive value
CRET	complete resection of enhancing tumor
FGS	fluorescence-guided surgery
iMRI	intraoperative Magnetic Resonance Imaging
EMEA	European Medicines Agency
